# Supplementary material for: Influence of Alcohol Consumption on the Development of Erosive Esophagitis in Both Sexes: A Longitudinal Study
Source: Nutrients. 2022 Nov 10;14(22):4760. doi: 10.3390/nu14224760 (PMC9697246; doi:10.3390/nu14224760)
Supplement: Supplementary file 1 [file nutrients-14-04760-s001.zip › nutrients-1957601-supplementary.pdf]

Supplementary Information S1

Evaluation of *Helicobacter pylori* infection

Serological *H. pylori* status was assessed using an enzyme-linked immunosorbent assay (Eiken Chemical, Tokyo, Japan). The seropositive antibody titer threshold for *H. pylori* infection was set to at least 3 U/mL. An increase in Δ13C values of >2.5‰ in the urea breath test (UBT) indicated positive results. Non-*H. pylori* infection was defined as follows: (1) patients *with H. pylori* antibody seronegativity and/or UBT negativity and no endoscopically atrophic gastritis; (2) those who did not undergo *H. pylori* antibody test or UBT, had no history of eradication therapy, had endoscopically regular arrangement of collecting venules, and the absence of atrophic gastritis; and (3) those with a history of eradication therapy and confirmed for the absence of *H. pylori* using UBT. We instituted a strict definition of non-*H. pylori* infection; therefore, individuals not fulfilling the above criteria were defined to have *H. pylori* infection.

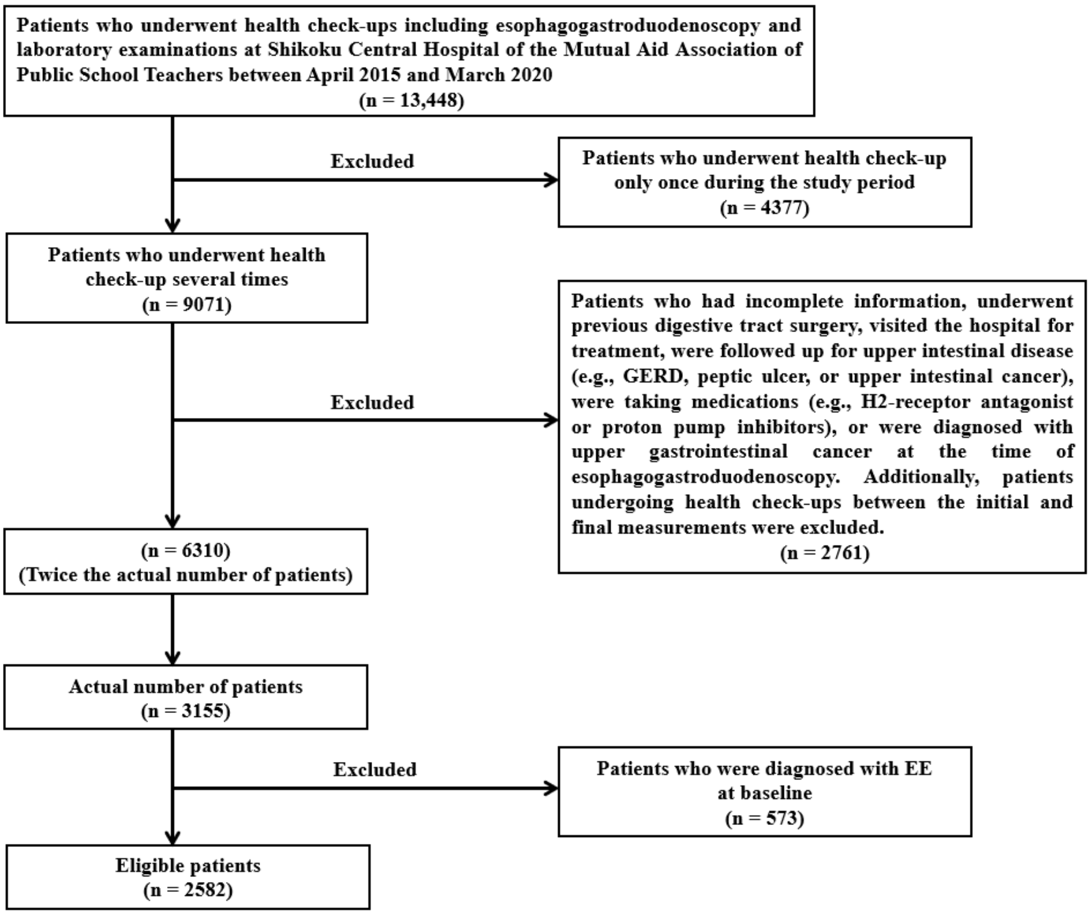

Figure S1. Participant flow diagram of individuals undergoing health check-ups. EE, erosive esophagitis; GERD, gastroesophageal reflux disease.

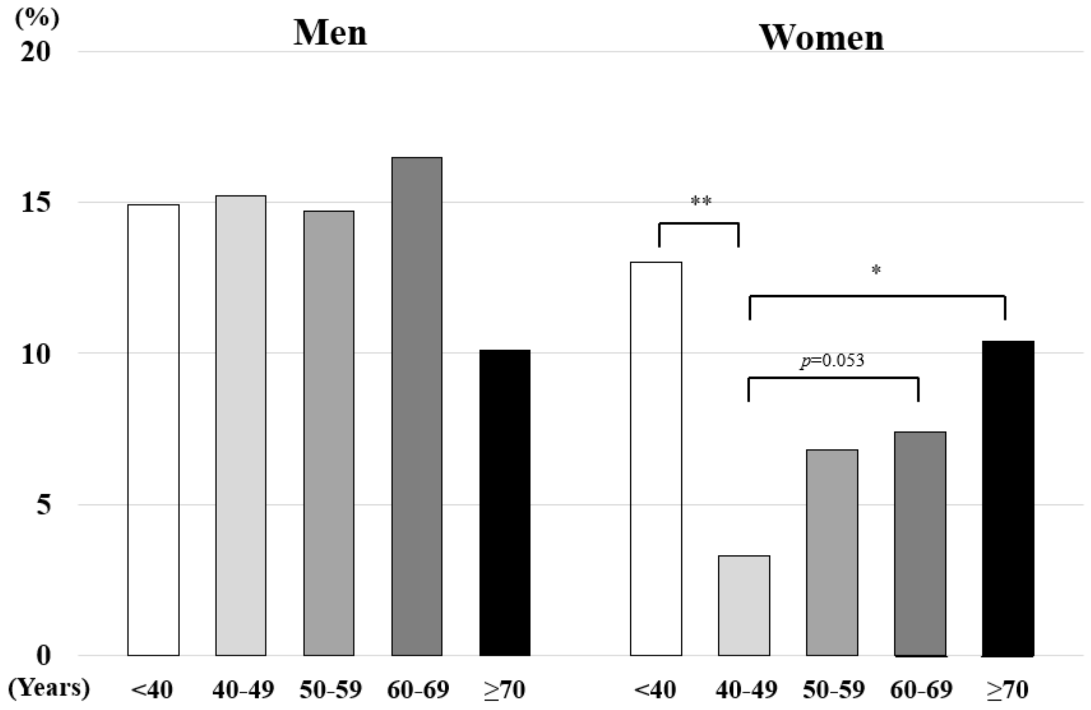

Figure S2. Comparison across age groups of the prevalence of EE development according to sex**.**
*p<0.05, **p<0.01. EE, erosive esophagitis.

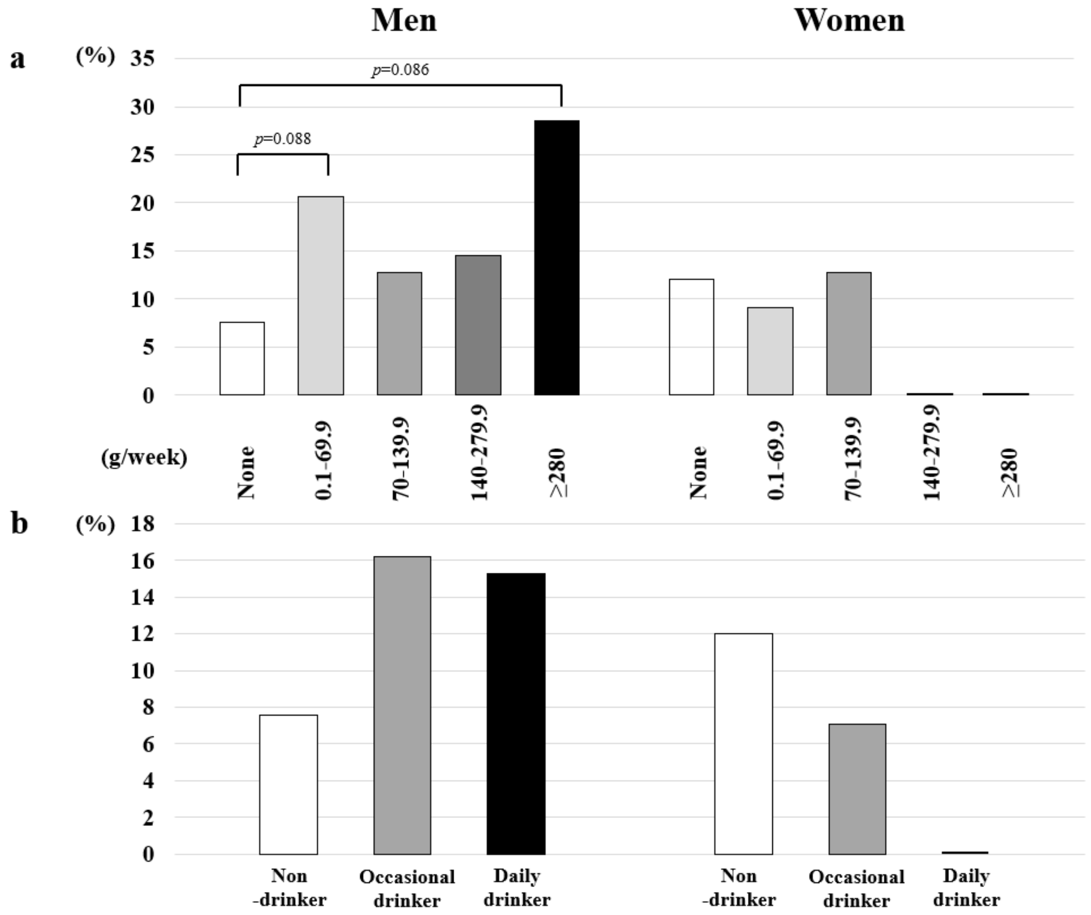

Figure S3. (**a**) Prevalence of severe EE according to drinking quantity categories and sex. EE, erosive esophagitis. (**b**) Prevalence of severe EE according to drinking frequency categories and sex. EE, erosive esophagitis.

Table S1 Relationship between alcohol quantity and EE severity according to sex (n = 290)

Men (n = 211)		Severity of EE			
Alcohol quantity (g/week)		Grade A	Grade B	Grade C	Grade D
None	52 (100%)	48 (92.3%)	2 (3.8%)	2 (3.8%)	0 (0%)
0.1–69.9	29 (100%)	23 (79.3%)	6 (20.7%)	0 (0%)	0 (0%)
70–139.9	47 (100%)	41 (87.2%)	5 (10.6%)	1 (2.1%)	0 (0%)
140–279.9	76 (100%)	65 (85.5%)	9 (11.8%)	1 (1.3%)	1 (1.3%)
≥280	7 (100%)	5 (71.4%)	2 (28.6%)	0 (0%)	0 (0%)
Women (n = 79)		Severity of EE			
Alcohol quantity (g/week)		Grade A	Grade B	Grade C	Grade D
None	50 (100%)	44 (88.0%)	6 (12.0%)	0 (0%)	0 (0%)
0.1–69.9	11 (100%)	10 (90.9%)	1 (9.1%)	0 (0%)	0 (0%)
70–139.9	6 (100%)	41 (87.2%)	5 (10.6%)	0 (0%)	0 (0%)
140–279.9	11 (100%)	11 (100%)	0 (0%)	0 (0%)	0 (0%)
≥280	1 (100%)	1 (100%)	0 (0%)	0 (0%)	0 (0%)

EE, erosive esophagitis

Table S2 Relationship between drinking frequency and the severity of EE according to sex (n = 290)

Men (n = 211)		Severity of EE			
Drinking frequency		Grade A	Grade B	Grade C	Grade D
Non-drinking	52 (100%)	48 (92.3%)	2 (3.8%)	2 (3.8%)	0 (0%)
Occasional drinking	74 (100%)	62 (83.8%)	12 (16.2%)	0 (0%)	0 (0%)
Daily drinking	85 (100%)	72 (84.7%)	10 (11.8%)	2 (2.4%)	1 (1.2%)
Women (n = 79)		Severity of EE			
Drinking frequency		Grade A	Grade B	Grade C	Grade D
Non-drinking	50 (100%)	44 (88.0%)	6 (12.0%)	0 (0%)	0 (0%)
Occasional drinking	14 (100%)	13 (92.9%)	1 (7.1%)	0 (0%)	0 (0%)
Daily drinking	15 (100%)	15 (100%)	0 (0%)	0 (0%)	0 (0%)

EE, erosive esophagitis

Table S3 Odds ratio for the development of EE in each category of alcohol quantity and other factors

		Men			Women		
		Overall	<50 years	≥50 years	Overall	<50 years	≥50 years
Age	(years)	1.017 (1.000–1.035)	1.052 (0.992–1.116)	1.035 (1.007–1.065)	1.026 (0.990–1.063)	0.975 (0.865–1.099)	1.057 (1.009–1.108)
BMI	(kg/m²)	1.094 (0.994–1.203)	0.999 (0.843–1.184)	1.140 (1.011–1.285)	1.045 (0.915–1.193)	0.977 (0.759–1.259)	1.077 (0.919–1.262)
WC	(cm)	0.991 (0.956–1.029)	1.045 (0.975–1.120)	0.969 (0.928–1.012)	1.005 (0.958–1.054)	1.007 (0.924–1.096)	1.001 (0.947–1.059)
Current smokers		1.373 (0.998–1.888)	1.249 (0.722–2.159)	1.449 (0.976–2.151)	1.007 (0.283–3.591)	(–)	1.953 (0.474–7.994)
Drinkers		1.252 (0.907–1.726)	2.312 (1.171–4.564)	1.009 (0.694–1.466)	1.078 (0.666–1.747)	1.764 (0.736–4.231)	0.853 (0.461–1.581)
Alcohol quantity	(g/week)						
	0.1–69.9	0.971 (0.609–1.547)	1.154 (0.461–2.885)	0.989 (0.572–1.709)	0.692 (0.353–1.356)	1.072 (0.328–3.505)	0.589 (0.253–1.370)
	70–139.9	1.443 (0.954–2.182)	2.878 (1.302–6.361)	1.134 (0.682–1.883)	0.911 (0.375–2.210)	1.873 (0.481–7.299)	0.619 (0.182–2.110)
	140–279.9	1.255 (0.875–1.799)	2.666 (1.283–5.542)	0.945 (0.621–1.438)	3.040 (1.504–6.146)	3.074 (0.940–10.05)	3.149 (1.230–8.064)
	≥280	2.014 (0.848–4.787)	4.549 (1.228–16.85)	1.227 (0.354–4.255)	13.26 (1.735–101.4)	30.87 (3.693–258.1)	(–)
Regular exercise		1.028 (0.754–1.400)	1.330 (0.777–2.277)	0.897 (0.609–1.321)	1.005 (0.590–1.714)	0.282 (0.035–2.279)	1.146 (0.629–2.088)
Eating before going to bed		1.228 (0.917–1.643)	1.256 (0.759–2.078)	1.269 (0.874–1.841)	0.851 (0.492–1.472)	0.943 (0.381–2.334)	0.776 (0.371–1.621)
Custom of having breakfast		0.857 (0.535–1.373)	0.634 (0.301–1.338)	1.048 (0.569–1.929)	0.597 (0.233–1.528)	0.360 (0.088–1.475)	0.724 (0.202–2.594)
Hypertension		1.044 (0.757–1.441)	0.719 (0.412–1.254)	1.293 (0.853–1.959)	1.140 (0.683–1.903)	1.820 (0.728–4.550)	0.934 (0.496–1.760)
Dyslipidemia		0.883 (0.649–1.201)	0.689 (0.385–1.231)	0.985 (0.681–1.424)	1.397 (0.803–2.431)	2.081 (0.731–5.924)	1.226 (0.645–2.331)
IGT		1.373 (1.010–1.882)	1.480 (0.849–2.578)	1.263 (0.861–1.853)	1.204 (0.728–1.992)	1.804 (0.690–4.716)	1.070 (0.599–1.911)
H. pylori positivity		0.266 (0.151–0.471)	0.073 (0.011–0.500)	0.358 (0.193–0.665)	0.242 (0.076–0.771)	0.403 (0.068–2.382)	0.190 (0.046–0.790)

Data are presented as odds ratio and 95% confidence interval. (-) means including the absence of participants in category.

BMI, body mass index; EE, erosive esophagitis; *H. pylori*, *Helicobacter pylori*; IGT, impaired glucose tolerance; WC, waist circumference
